# Prevalence of excess weight and associated socio-demographic factors among postmenopausal women: A population-based study in Ghana

**DOI:** 10.4102/phcfm.v15i1.3781

**Published:** 2023-03-09

**Authors:** Isaac Mensah Bonsu, Corlia Brandt, Adedayo T. Ajidahun, Monday O. Moses, Hellen Myezwa

**Affiliations:** 1 Department of Physiotherapy, School of Therapeutic Sciences, University of the Witwatersrand, Johannesburg, South Africa; 2 Department of Physiotherapy and Sports Science, College of Health Sciences, Faculty of Allied Health Sciences, Kwame Nkrumah University of Science and Technology, Kumasi, Ghana

**Keywords:** desirable weight, body mass index, obesity, postmenopausal, waist-to-height ratio, waist-to-hip ratio, prevalence

## Abstract

**Background:**

Excess weight (obesity and overweight) is a pervasive condition that is considered a global epidemic and a threat to public health. Furthermore, numerous changes in fat deposits occur with the advent of menopause, leading to a change in the distribution of body fat. Knowledge of sociodemographic factors and prevalence can inform the effective management of these women.

**Aim:**

This study aimed to investigate the prevalence of excess weight among postmenopausal women in Ghana’s Bono East (Techiman) region.

**Setting:**

This study was conducted in Bono East regional capital, Techiman, Ghana.

**Methods:**

This is a cross-sectional study conducted over 5 months at Bono East regional capital, Techiman in Ghana. Anthropometric parameters such as body mass index (BMI), waist-to-hip ratio (WHR) and waist-to-height ratio (WHtR) were obtained using physical measurements while socio-demographic data were gathered using questionnaires. Data analysis was performed using IBM SPSS 25.

**Results:**

The mean age of the 378 women who participated in the study was 60.09 ± 6.24 years. Body mass index, WHtR and WHR indicated excess weight of 73.2%, 91.8% and 91.0%, respectively. Education and ethnicity were predictors of excess weight (WHR). Women of the Ga tribe with high school education have 4.7- and 8.6-times increased odds of having excess weight.

**Conclusions:**

There are higher prevalence rates of excess weight (obesity and overweight) among postmenopausal women using BMI, WHtR and WHR. Education and ethnicity are predictors of excess weight.

**Contribution:**

The study’s findings can be used to develop interventions that focus on addressing excess weight in postmenopausal women within the Ghanaian context.

## Introduction

One of the major factors of poor health is excess weight (obesity and overweight),^1^ exceeding other causes such as undernutrition and infectious diseases.^2^ Obesity has been more widespread over the world in the last 50 years,^3^ and the incidence of excess body weight among women has reached epidemic proportions.^4^ The onset of menopause in middle age is linked to an increased tendency to gain weight.^5,6^ It is projected that middle-aged (40–59) women have a greater obesity rate, (42.1%) than women aged 20–39 (34.4%).^7^ In Ghana, a survey by Agbeko and Akwasi^8^ found that the rates of excess weight were shown to be positively linked with age and ranged from 14% for women aged 15–24 to 43% for persons aged 35–44 years old.

Excess weight is the outcome of a complex interaction of different genetic factors derived from a person’s eating behaviours, energy expenditure factors and physical activity.^3^ Also, with the onset of menopause, multiple changes in fat deposition occur, resulting in a shift in body fat distribution. Oestrogen deficiency has a deleterious influence on fat metabolism, resulting in abdominal obesity.^9,10^ Weight gain in menopause is intimately linked to age, lifestyle and behavioural variables such as physical inactivity and an increase in food consumption.^3^

Obesity is a key public health problem that significantly raises the risk of osteoarthritis, cardiovascular disease, dementia and several cancers among other diseases, thereby contributing to a decline in both quality of life and life expectancy.^11^ Obesity is also associated with unemployment, social disadvantages and decreased socioeconomic production, putting the economy under pressure.^3^ Several studies have tried to interpret the factors related to weight gain during menopause,^12,13^ but few population-based studies have examined the demographical factors associated with the prevalence of obesity and overweight in postmenopausal women, particularly in sub-Saharan African countries such as Ghana.

Multiple measures are required to decrease the burden of excess weight, including individual interventions as well as changes in the society and environment.^14^ As a result, gaining a better understanding of regional disparities in obesity prevalence and trends could aid in identifying societal causes of obesity and advising on the most viable intervention techniques. Given this, the study sought to investigate the prevalence of excess weight among postmenopausal women in Ghana’s Bono East (Techiman) region, as well as the socio-demographic factors that influence it.

## Research methods and design

### Study site and design

A 5-month cross-sectional study was undertaken in Ghana’s Bono East regional capital, Techiman, from October 2019 to February 2020.

### Study population and sampling strategy

The population consisted of three geopolitical zones in Ghana: northern, middle and southern. Sequentially, a zone, region and district were selected by simple random sampling. The study participants were recruited via public invitation (such as information centres and announcements at churches and mosques). Women’s fellowships, women’s groups, churches and mosques were randomly selected throughout the district. Participants who responded to the invitations were recruited from their households, churches, mosques and communities. Using Ghana’s 2010 population and housing census, the sample frame consisted of postmenopausal women aged 45 and older.^15^ Using the sample size formula for cross-sectional studies in an unknown population, a sample size of 358 was estimated.^16^ The final sample size was 393 including a 10% fall-out rate. The figure was derived from the rate of obesity among Ghanaian postmenopausal women (37.1%, *p* = 0.37),^17^ with a margin of error of 0.05 and a confidence interval of 95%. The sample size formula for cross-sectional studies was:


(Z2)2P(1−P)d^2 [Eqn 1]

### Criteria for inclusion and exclusion

The study included postmenopausal Ghanaian women over the age of 45 who were not on any weight-loss therapy and did not have any physical or mental disabilities. Women who are not Ghanaian and had not resided in Ghana for the previous three years before the study were not eligible.

### Data collection

#### Questionnaire

The self-developed questionnaire obtained demographic information such as age, educational background, ethnic origin, marital status, parity and religion. Information regarding participants’ last menstrual period was also obtained (Table 1). The last menstrual period was defined as the final menstrual period and is confirmed when a participant has not had her period for 12 consecutive months.

**TABLE 1:** Demographic characteristics of the participants (*N* = 378).

Demographics	Mean	s.d.	*n*	%
**Age in years**	60.09	6.24	-	-
**†Age group (years), *N* = 369**				
≤ 50	-	-	24	6.5
51–60	-	-	180	48.8
61–70	-	-	150	40.7
> 70	-	-	15	4.2
**†Religion, *N* = 368**				
Christian	-	-	192	52.2
Muslim	-	-	151	41
Others	-	-	25	6.8
**†Marital status, *N* = 369**				
Married	-	-	130	35.2
Widowed	-	-	153	41.5
Single	-	-	34	9.2
Divorced	-	-	52	14.1
**†Ethnic origin, *N* = 366**				
Northern people	-	-	137	37.4
Akan people	-	-	148	40.4
Ga people	-	-	42	11.5
Voltarian people	-	-	34	9.3
Others	-	-	5	1.4
**†Educational background, *N* = 363**				
Primary	-	-	155	42.7
High school	-	-	97	26.7
Tertiary	-	-	68	18.8
Others	-	-	43	11.8
**†Parity, *N* = 292**				
< 3	-	-	41	14
4–7	-	-	174	59.6
> 7	-	-	77	26.4

s.d., standard deviation.

† , Missing data.

#### Measurement of weight, height and body mass index measurement

An analogue balance stadiometer (SECA 285) was used to assess body weight and height (to the nearest 0.5 kg and 0.5 cm, respectively). The participants’ posture was measured to ensure that essential postural points were constantly positioned, such as the feet being together, knees touching, buttocks, scapulae and head in contact with the vertical backboard, the back against the board and heels at the base of the vertical board. The arms of the participant hang freely by the sides of the trunk with palms towards the thighs. The ratio of body weight to height squared was used to calculate body mass index (BMI). Using the World Health Organization (WHO) criteria, BMI < 18.5 kg/m^2^ underweight; BMI 18.5 kg/m^2^-24.9 kg/m^2^ normal weight; BMI 25.0 kg/m^2^–29.9 kg/m^2^ overweight; and > 30.0 kg/m^2^ obese.^18^ Excess weight was defined as a participant with a BMI ≥ 25.0 kg/m^2^.

#### Measurement of waist circumference, hip circumference, waist-to-hip ratio and waist-to-height ratio

With the participant in a standing position (with feet together), the inelastic tape was used to measure waist and hip circumference (to the nearest 0.1 cm) as the smallest girth around the waist and the region of maximum girth around the buttocks – (the trochanteric region) for hip circumference.^19^ The waist-hip ratio (Eqn 2) and the waist-to-height ratio (WHtR) (Eqn 1) were determined using the waist-to-hip circumference equations:


$$\text{Waist-to-hip ratio}=\frac{\text{Waist circumference }(\text{cm})}{\text{Hip circumference }(\text{cm})}$$ [Eqn 2]

$$\text{Waist-to-height ratio}=\frac{\text{Waist circumference }(\text{cm})}{\text{Height circumference }(\text{cm})}$$ [Eqn 3]

Waist circumference (WC) >88 cm and waist-to-hip ratio (WHR) > 0.8^20^ were used to indicate abdominal obesity (WHR), while WHtR was defined as normal: < 0.4, overweight: 0.4–0.5, obese: 0.5–0.6 and morbidly obese: > 0.6.^21,22^

### Data analysis

Microsoft Excel was used to enter and clean the data, then it was exported to IBM SPSS (version 25.0) for analysis. In order to minimize measurement inconsistencies, the authors double-checked the data that were captured on an Excel spreadsheet. The descriptive statistics included frequencies, percentages, means and standard deviation to summarize all variables. The *K*-test was used to determine the differences between proportions. Multivariate logistic regression with the Enter method was used to assess the association between demographics and the various indexes of weight gain. The dependent variable was obesity, and the independent variables were socio-demographic and clinical characteristics. The multivariate logistic regression model was adjusted for age. Statistical significance was set at *p* < 0.05 for all comparisons. Missing data were treated as missing.

### Ethical considerations

Ethical approval for this study was obtained from the Human Research Ethics Committee at the University of the Witwatersrand, South Africa (Ref no. M190467). In Ghana, permission was also obtained from the Committee on Human Research, Publication and Ethics of Kwame Nkrumah University of Science and Technology, Kumasi (Ref No. 596/19).

## Results

A total of 378 postmenopausal women who participated in this study ranged in age from 45 to 80 years old, with a mean age of 60.09 ± 6.24 years.

### Prevalence of excess weight among participants

**FIGURE 1:**
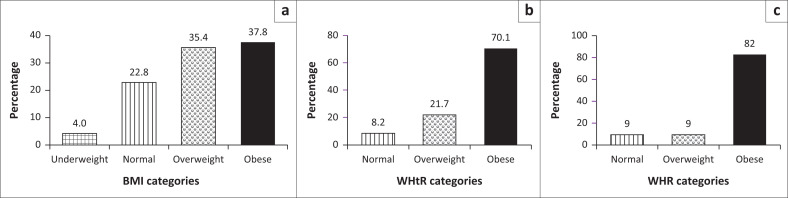
Prevalence of excess weight among study participants stratified by (a) body mass index categories, (b) waist-to-height ratio categories (c) waist-to-hip ratio categories.

Excess weight is described as overweight and obese on all three indices (BMI, WHR and WHtR), whereas desirable weight is defined as those with normal body weight using all three indices. Using the WHO cut-offs of excess weight as a reference, 37.8% of the participants were obese, 35.4% overweight, 22.8% had normal weight and 4.0% were underweight (Figure 1a). Figure 1b shows that 70.1% of the participants were obese, while 8.2% had a normal WHtR. According to the WHR, 82.0% were obese, and 9.0% were overweight (Figure 1c).

**FIGURE 2:**
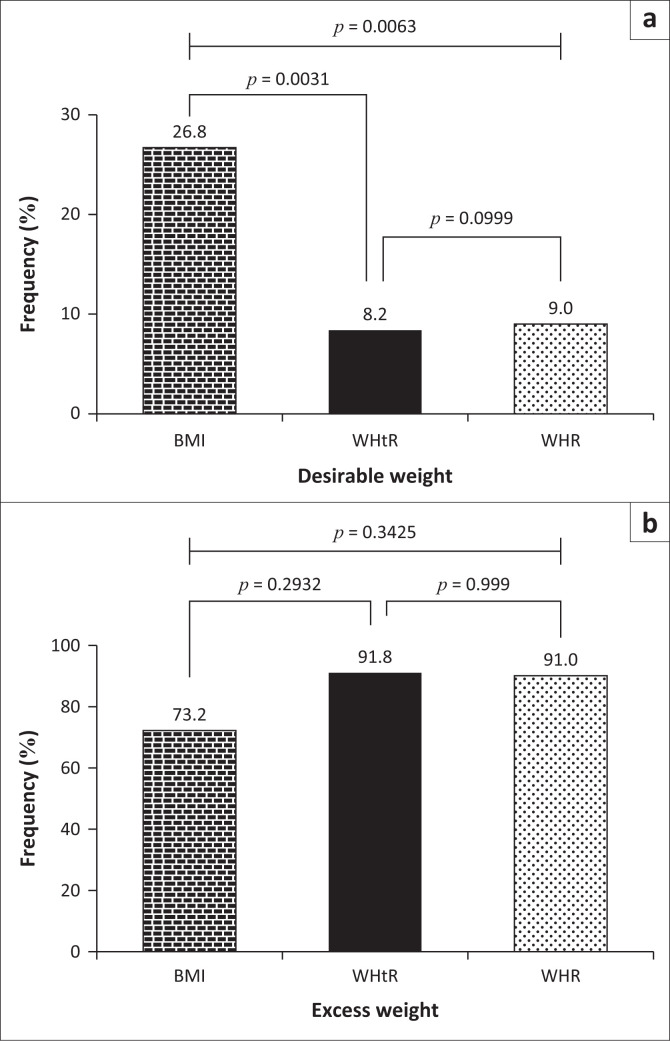
Distribution of body mass index, waist-to-height ratio and waist-to-hip ratio by desirable (A) and excess weight (B).

Although there was no significant difference (*p* = 0.999) between the proportion of study participants with a desirable weight for WHtR and WHR, the BMI, WHtR and WHR findings revealed that 26.8%, 8.2% and 9.0% of the sample had a desirable weight (Figure 2a). The distribution of study participants with desirable weight categorized by BMI was significantly higher than that of WHtR (*p* = 0.0031) and WHR (*p* = 0.0063). Results indicated that 73.2%, 91.8% and 91.0% of the participants had excess weight using BMI, WHtR and WHR, respectively, and there was no substantial difference in excess weight among the anthropometric indices (BMI, WHtR or WHR) measured (Figure 2b).

Table 2 shows a multivariate binary logistic regression analysis of socio-demographic factors for the odds ratio of developing obesity among study participants. Postmenopausal women of the Ga tribe compared with the northern tribe had 4.7 times increased odds of obesity characterized by the WHR criterion. Also, postmenopausal women who were high school graduates compared with those who only had primary school education, had 8.6 times increased odds of being obese characterized by the WHR criterion. Conversely, participants who have given birth 4–7 times were at 20% decreased odds of obesity. Age group, marital status and postmenopausal stage were independent factors that were not associated with excess weight.

**TABLE 2:** Multivariate binary logistic regression analysis, indicating the odds ratio for demographic risk factors for obesity among study participants.

Demographics	BMI-obesity		WHR-obesity		WHtR-obesity	
	aOR	95%CI	aOR	95%CI	aOR	95%CI
**Age group (years)**						
≤ 50	1		1	-	1	-
51–60	0.4	0.1–2.0	-	-	0.6	0.1–3.3
61–70	0.3	0.1–1.9	-	-	0.5	0.1–3.3
> 70	-	-	0.8	0.2–1.6	0.2	0.0–7.2
**Religion**						
Christian	1	-	1	-	1	-
Muslim	1.8	0.8–4.3	0.9	0.2–3.0	1.1	0.5–2.7
Others	-	-	0.2	0.0–1.7	1.1	0.2–6.9
**Marital**						
Married	1	-	1	-	1	-
Widow	1.1	0.4–2.8	1.1	0.3–3.7	1.4	0.6–3.4
Single	1.1	0.2–5.0	3.5	0.4–32.7	0.7	0.2–2.9
Divorced	2.3	0.7–7.5	2.5	0.4–15.5	2.7	0.7–10.7
**Ethnic origin**						
Northern people	1	-	1	-	1	-
Akan people	1.4	0.6–3.6	0.1	0.0–0.7	3.2	1.2–8.51
Ga people	3.1	0.8–12.4	4.7	1.1–20.6*	2.0	0.5–7.9
Voltarian people	1.0	0.2–4.6	0.6	0.1–4.4	1.2	0.3–4.8
Others	-	-	-	-	-	-
**Educational background**						
Primary	1	-	1	-	1	-
High school	0.8	0.3–1.8	8.6	2.0–36.9*	1.3	0.5–3.2
Tertiary	0.4	0.1–1.3	1.0	0.3–2.7	1.8	0.5–6.1
Others	0.1	0.0–0.6	1.9	0.4–8.9	1.2	0.4–4.0
**Gravidity**						
< 3	1	-	1	-	1	-
4–7	0.8	0.3–2.0	0.2	0.0–0.9	0.8	0.3–2.2
> 7	1.5	0.4–5.5	0.5	0.1–3.5	1.9	0.5–7.7
**Postmenopausal stage (years)**						
≤ 5	1	-	1	-	1	-
6–10	1.0	0.4–2.4	0.7	0.2–2.4	0.8	0.3–2.0
11–15	0.9	0.2–5.1	0.5	0.1–3.2	0.9	0.2–5.2
> 15	4.0	0.3–61.0	0.2	0–4.4	1.5	0.1–23.1

aOR, age adjusted odds ratio; CI, confidence interval; BMI, body mass index; WHR, waist-to-hip ratio; WHtR, waist-to-height ratio.

Prevalence of excess weight.

* *p* < 0.05 = statistically significant.

## Discussion

Using BMI, WHtR and WHR, the study found a high prevalence rate of excess weight among the participants of 73.2%, 91.8% and 91.0%, respectively. The stratified study participants using the anthropometric indices (BMI, WHtR or WHR) showed no significant differences. Postmenopausal women of the Ga tribe compared with the northern tribe had 4.7 times increased odds of excess weight indicated by WHR criterion. Women from the northern tribe are known to engage in non-mechanized farming, which is the major occupation compared with the other ethnic groups. Again, cultural settings in the northern tribe are such that women are mostly responsible for all household activities such as washing, cooking sweeping and these are forms of physical activities that prevent excess weight to thrive. Women from this tribe are mostly stout, which shows an inverse BMI–height association.^23^ In addition, the standard of living of most people from the north is low; they eat what they grow. Their diet is free from chunks of canned, junk and fatty foods, which are a major contributor to excess weight.

Furthermore, participants who were high school graduates compared with primary school had 8.6 times increased odds of being obese indicated by the WHR criterion. Conversely, participants who have given birth 4–7 times had 20% decreased odds of excess weight. Age group, religion, marital status and postmenopausal stage were not independent factors associated with excess weight.

The postmenopausal stage (years) was not significantly associated with excess weight among participants. Menopause is often a source of anxiety for women.^24^ One of the most significant is the dread of gaining weight.^25^ Obesity and metabolic syndrome are three times more common in women throughout this stage of their lives than they are before menopause.^26^ The body composition of postmenopausal women alters to more fat and less muscle, which slows the rate at which the body metabolizes biomolecules.^27^ The changes in body composition are associated with weight gain, particularly around the abdominal region, which contributes to metabolic abnormalities and a higher prevalence of metabolic syndrome.^27^

Weight gain was found to be prevalent among participants. According to the BMI, 37.8% of the participants were obese, followed by overweight (35.4%), normal (22.8%) and underweight (4.0%). Gravena and Brischiliari^28^ found a similar prevalence rate of obesity (35.5%) in postmenopausal Brazilian women using BMI in their investigation of excess weight and abdominal obesity. Abdominal obesity (63.3%) was observed in the same study using WC.^28^ However, they reported a greater prevalence rate of overweight (72.6%) than this study, which could be because of the age difference, because the mean age was 58.7 ± 5.7 years compared with 60.09 ± 6.24 years for this study.

The natural history of the menopausal transition was explored by the Study of Women’s Health Across the Nation (SWAN), which found that WC increased with age and accelerated after menopause.^29^ According to Stevens and Katz^30^ WC and WHR are controlled by body weight, body composition and fat distribution and have relationships and increase with age. This could be because of hormonal and behavioural factors in their bodies, both of which contribute to their excessive weight gain.^9,10^

According to WHR findings, 82.0% were obese, 9.0% were overweight and 9.0% were normal. Also, 70.1% were obese and 8.2% were normal according to the WHtR. This trend is evident in similar populations elsewhere,^31^ which established 5.6% overweight and 93.5% obese according to WHR among postmenopausal Ghanaian women. Furthermore, using the WHtR, 19.8% of women in Arthur et al.’s study were normal, while 83.2% were obese.^31^ Although Kow Nanse Arthur et al.,^31^ reported greater prevalence rates than this study, there is positive congruence between the two investigations, suggesting that WHR may be a suitable anthropometric indicator for assessing obesity in women.^31,32^

In addition, Kissebah and Krakowah found that WHR presents significant indices of abdominal fat accumulation and has a better link with a higher risk of diseases than BMI alone.^33^ The WHO acknowledges that BMI can be used to estimate the prevalence of obesity in a population, but it does not account for the wide range of body fat distribution.^34^

There was no significant difference in proportions for WHtR and WHR with a desirable weight gain, which was an interesting finding. Waist-to-height ratio and WHR, on the other hand, had a much larger distribution among study participants with a desirable weight based on their BMI than WHtR and WHR. Body mass index, WHtR and WHR recorded percentages of excess weight of 73.2%, 91.8% and 91.0%, respectively, among the general study participants, and there was no significant difference among the stratified study participants. Al-Safi and Polotsky^35^ observed considerably high excess weight gain in most postmenopausal women, which is not similar to the higher proportion of BMI, WHtR and WHR in this study.

Excess weight prevalence rates in this study differed from those in previous studies, which could be attributable to age variations. The average age of the participants in this study was 60.09 ± 6.24 years, which was slightly higher than the average age (57.25 ± 0.80) of participants in the study conducted by Kow Nanse Arthur et al.^31^ The increases in WC with age, particularly during the postmenopausal stage, were because of weight gain, although WC increases have also been documented in the absence of weight gain.^36,37^ Participants aged ≤ 50 had a higher probability of being obese in all anthropometric parameters, although not substantially higher than other age groups.

Study participants who acquired high school education compared with primary school had 8.6 times increased odds of being obese, as characterized by WHR criterion. Changing nutritional trends and exposure to Western lifestyles might be contributing factors to these trends, which have health complications. Examples could include following a Western diet, a sedentary lifestyle and physical inactivity. The finding of this study is consistent with a survey conducted by Ziraba et al.,^38^ which revealed that women with secondary or higher education were 60% more likely to gain excess weight (overweight or obese) compared with a similar population with no education (*p* < 0.01). Ofori-Asenso^39^ substantiate the claim that, when compared with less educated and illiterate people, Ghanaians with tertiary education have the highest prevalence of obesity. This may conclude the idea that larger body sizes may be sensitive and affluence in many Ghanaian communities^17^; nevertheless, it may also affirm the fact that increasing affluence may be associated with an increase in larger portion sizes.^39^

The WHR revealed that postmenopausal Ga women were more likely to be obese. This could be driven by the societal acceptability of weight gain in women in this tribe. The earlier study of women’s perceptions and experiences of body weight in the Ga East District by Aryeetey^40^ described weight gain as socially admired and presentable, and it is expected when individuals or families experience improved livelihoods. Again, most women from the Ga tribe are traders and they have access to unlimited food resources in the market that are energy dense with a consequent reduction in energy expenditure.^41^ Polygamous marriage is accepted among the Ga tribe. As a result of this, women who find themselves in this marriage mostly exhibit to their co-wives their comfortability and happiness in the marriage by being fat to demonstrate that the husband is taking good care of them.^42^ This could be contributing to the tribe’s growing excess weight gain pandemic.This finding is consistent with the outcomes of a study by Ofori-Asenso,^42^ which found that overweight and obesity are prevalent in Greater Accra, which largely has Ga tribe as inhabitants and the variations in excess weight prevalence among the ethnic groups are thought to mostly reflect differences in social behaviours including meal preparation, work patterns and perception of body size.

Although the results of this study are comparable to those of prior investigations, the cross-sectional design prevents causality from being established. The lack of evaluation of the participants’ nutritional status as part of the factors of nutritional state in older age^34^ could be a study limitation. As the anthropometric measurements were limited to women who had lived in Ghana for the previous 3 years, the female population included in this study may not be typical of the postmenopausal women population.

## Conclusion

Excess weight was common among postmenopausal Ghanaian women in this study. These results may have important implications for women’s health because excess weight can lead to a risk of chronic diseases, higher healthcare costs, and a reduction in quality of life and overall well-being. However, factors that influence excess weight, including social influences, lifestyle, and levels of physical activity, should be studied and evaluated. Furthermore, it will be useful to explore weight management practices among postmenopausal women. Exploring indigenous practices may be useful for the future management of weight gain.

## Contributions to research

This study confirms a high prevalence of postmenopausal women with excess weight using BMI, WHtR and WHR. The higher prevalence of excess weight based on BMI, WHR and WtHR is associated with vascular and metabolic risk and this reinforces the importance of strategies to address overweight and obesity issues to prevent complications. Postmenopausal women from the Ga tribe and high school graduates have a higher chance of being overweight or obese characterized by WHR criterion. This study found that the prevalences of excess weight were higher for postmenopausal women who had four to seven children. Future research should focus on developing intervention programmes for postmenopausal women to lose body weight and improve health.
